# Contaminants of Emerging Concern in Bats from the Northeastern United States

**DOI:** 10.1007/s00244-015-0196-x

**Published:** 2015-08-06

**Authors:** Anne L. Secord, Kathleen A. Patnode, Charles Carter, Eric Redman, Daniel J. Gefell, Andrew R. Major, Daniel W. Sparks

**Affiliations:** U.S. Fish and Wildlife Service, 3817 Luker Road, Cortland, NY 13045 USA; U.S. Fish and Wildlife Service, 110 Radnor Road, Suite 101, State College, PA 16801 USA; TestAmerica, 3275 S. Tioga Way, Las Vegas, NV 89117 USA; TestAmerica, 880 Riverside Parkway, West Sacramento, CA 95605 USA; U.S. Fish and Wildlife Service, 70 Commercial St., Suite 300, Concord, NH 03301 USA; U.S. Fish and Wildlife Service, 620 S. Walker St., Bloomington, IN 47403 USA

## Abstract

We analyzed bat carcasses (*Myotis lucifugus*, *M. sodalis*, *M. septentrionalis*, and *Eptesicus fuscus*) from the northeastern United States for contaminants of emerging concern (CECs) such as polybrominated diphenyl ethers (PBDEs), and pharmaceuticals and personal care products. The CECs detected most frequently in samples were PBDEs (100 %), salicylic acid (81 %), thiabendazole (50 %), and caffeine (23 %). Other compounds detected in at least 15 % of bat samples were digoxigenin, ibuprofen, warfarin, penicillin V, testosterone, and *N*,*N*-diethyl-meta-toluamide (DEET). The CECs present at the highest geometric mean wet weight concentrations in bat carcasses were bisphenol A (397 ng/g), ΣPDBE congeners 28, 47, 99, 100, 153, and 154 (83.5 ng/g), triclosan (71.3 n/g), caffeine (68.3 ng/g), salicylic acid (66.4 ng/g), warfarin (57.6 ng/g), sulfathiazole (55.8 ng/g), tris(1-chloro-2-propyl) phosphate (53.8 ng/g), and DEET (37.2 ng/g). Bats frequently forage in aquatic and terrestrial habitats that may be subjected to discharges from wastewater-treatment plants, agricultural operations, and other point and nonpoint sources of contaminants. This study shows that some CECs are accumulating in the tissue of bats. We propose that CECs detected in bats have the potential to affect a number of physiological systems in bats including hibernation, immune function, and response to white-nose syndrome, a fungal disease causing population-level impacts to bats.

**Electronic supplementary material:**

The online version of this article (doi:10.1007/s00244-015-0196-x) contains supplementary material, which is available to authorized users.

For their body size, bats live longer than any other order of mammal (5 to >30 years) (Austad and Fischer 1991). The northeastern bats that are the subject of this article have high metabolic rates resulting in a high rate of food intake (Clark and Shore 2001). Their longevity, high metabolic rate, and their insectivorous diet increase their likelihood of exposure to bioaccumulating chemicals in the environment; studies have shown that environmental contaminants, particularly organochlorine pesticides, can accumulate in bats and cause mortality (Clark and Shore 2001). Bats may be more susceptible than other mammals to the effects of low doses of bioaccumulative contaminants due to their annual life cycles requiring significant fat deposition followed by extreme fat depletion during hibernation or migration, at which time contaminants may be mobilized into the brain and other tissues (Clark and Shore 2001). Geluso et al. (1976) showed that dichlorodiphenyldichloroethylene (DDE) was increased in the brains of Mexican free-tailed bats (*Tadarida brasiliensis*) to lethal concentrations (37–330 mg/kg) after induced exercise intended to simulate migration. Reference bats and unexercised treatment bats had brain DDE concentrations of 1.1–17 and 10–95 mg/kg, respectively. In addition, fat metabolism and mobilization of lipid-borne xenobiotics by bats during hibernation is coincident with reduced immune function during torpor (Meteyer et al. 2012), thus potentially exposing bats to contaminants when they are immunocompromised.

The concentrations of legacy contaminants, such as organochlorine pesticides and polychlorinated biphenyls (PCBs), have decreased in bat tissues since the 1970s (Bayat et al. 2014). This positive environmental trend is countered by the proliferation in the environment of new classes of pesticides (*e.g.*, neonicotinoids, pyrethroids) and contaminants of emerging concern (CECs) such as detergents/surfactants, antibacterials, pharmaceuticals and personal care products (PPCPs), plasticizers, and polybrominated diphenyl ethers (PBDEs). PBDEs have been detected in wild mammals such as southern sea otters (*Enhydra lutris nereis*) and small cetacean species from Asian waters (Kajiwara et al. 2006; Kannan et al. 2007). PBDEs were reported in fat and brain tissue of little brown bats (*Myotis lucifugus*) from New York State, USA (Kannan et al. 2010). The mean PBDE concentrations in bat lipid were 685 and 620 ng/g wet weight in male and female bats, respectively. The mean brain PBDE concentration in these bats was 21 ng/g wet weight (Kannan et al. 2010).

Many of the non-PBDE CECs have been detected in surface water, sediment (Kolpin et al. 2002; Howard and Muir 2011; Reif et al. 2012), and, to a lesser extent, fish (Ramirez et al. 2009), wild birds (Oaks et al. 2004; Sherburne et al. 2013; Lazarus et al. 2015), and wild mammals (Richards et al. 2011). Information on non-PBDE CECs in bat tissues is lacking in the literature, although Park et al. (2009) modeled the exposure of bats (*Pipistrellus pipistrellus*) to 12 endocrine disrupting chemicals (EDCs) detected in insects at sewage-treatment plants used as foraging sites by bats. EDCs detected in insects included 17α-ethinylestradiol, butylated hydroxyl aniline, and bisphenol A. Based on their modeled EDC concentrations in bats, they suggested that detrimental effects of EDCs on foraging bats is plausible.

The route of exposure by which CECs move from primarily human and veterinary sources to bats can be postulated based on our knowledge of waste-disposal practices and bat feeding ecology. CECs are discharged into streams and other water bodies (Kolpin et al. 2002; Howard and Muir 2011; Reif et al. 2012) or applied to land (Sarmah et al. 2006). Compounds such as caffeine, salicylic acid, ciproflaxin and gemfibrozil have been reported in wastewater influent, effluent, and biosolids (Spongberg and Witter 2008). Agriculture is a confirmed source of antibiotics to the environment by way of their use in livestock medications and feed and their presence in animal wastes (Sarmah et al. 2006). Bats such as little brown bats (*M. lucifugus*) feed on a variety of insects that may accumulate CECs from aquatic and terrestrial environments (Anthony and Kunz 1977; Hanski 1987).Scientists in Scotland found that sewage-treatment filter beds supported high insect density and were important foraging sites for bats (Park and Cristinacce 2006). Aerial invertebrates collected there contained greater concentrations of estrogenic compounds, such as 17α-ethinylestradiol and butylated hydroxyl aniline, than invertebrates collected at reference sites, thus providing a plausible direct link between CECs in sewage-treatment plant effluent and insects eaten by bats (Park et al. 2009).

CECs may exacerbate white-nose syndrome (WNS), a disease found in bats caused by the fungal pathogen *Pseudogymnoascus destructans*. Antibacterials and antibiotics may directly interfere with the fungal-bacterial balance in bats that are coping with WNS (Cornelison et al. 2014; Hoyt et al. 2015). Alterations in central nervous system signaling by adenosine antagonists, such as caffeine (Jinka et al. 2011) or prostaglandin imbalance from nonsteroidal anti-inflammatory drugs (NSAIDs) (Takahata et al. 1996; Prendergast et al. 2002; Arnold et al. 2012), may promote the frequent arousals observed in WNS bats (Reeder et al. 2012). Pharmaceuticals such as ibuprofen or diclofenac and the antidepressant fluoxetine have been shown to influence the physiology of wild birds (Oaks et al. 2004; Bean et al. 2014) and possibly mammals (Simpson et al. 2011), thus reminding us of the potential risks associated with pharmaceuticals in the environment.

The goal of this study was to provide preliminary data on CECs in bats in the northeastern United States and to propose recommendations on whether CECs should be given additional scrutiny for their potential impacts to bats.

## Materials and Methods

### Bat Carcass Collection and Processing

Forty-eight dead or moribund bats were collected by the United States Fish and Wildlife Service (USFWS) or state personnel in New York, Pennsylvania, Vermont, Massachusetts, and New Hampshire, USA. Bats were collected as part of WNS-surveillance programs from hibernacula or homes or they were collected by the New York State Health Department (NYSDOH) as part of their rabies-surveillance program. Species collected were *M. lucifugus* (MYLU), *M. sodalis* (MYSO), *M. septentrionalis* (MYSE), and *Eptesicus fuscus* (EPFU). Many of the non-NYSDOH bats analyzed for CECs were believed to have died as a result of WNS, although no polymerase chain reaction analysis was performed to confirm the presence of *P. destructans*. No bats were killed for this study in an effort to minimize mortality among bat populations that are already severely stressed as a result of WNS. All bat carcass samples were collected from 2008 to 2010. Because bat carcasses were collected opportunistically, the interval between time of death and collection and their preservation by way of freezing was not always known. There may have been degradation of contaminants or moisture/lipid loss, which may have influenced concentrations of analytes.

All bat carcasses were kept frozen soon after collection and shipped to the analytical laboratory frozen on dry ice by way of next-day air delivery. Twenty-six bat carcass samples [8 from Pennsylvania (PA), 3 from Massachusetts (MA), 2 from New Hampshire (NH), 2 from Vermont (VT), and 11 from New York (NY)] were analyzed for 75 CECs (PPCPs and hormones) by TestAmerica Laboratories (see Supplementary Table 1 for complete list). Twenty-two additional individual bat carcasses (3 from PA, 2 from MA, 2 from NH, 1 from VT, and 14 from NY) were analyzed for PBDEs by Mississippi State Chemical Laboratory.

**Table 1:** Contaminants of emerging concern detected in northeastern United States bats

Analyte	Average LOD associated with detected analytes (ng/g)	No. bats with concentration >LOD	Geometric mean concentration of detects (ng/g)	Maximum concentration (ng/g)	Log k_ow_	Use
Pharmaceuticals (nonantibiotic)						
Digoxigenin	4.6	4/26	8.73	36.6	1.60	Cardenolide
Diltiazem	0.02	1/26	0.11	0.11	2.79	Antihypertensive, vasodilator
Gemfibrozil	0.03	2/26	0.65	0.75	4.77	Antilipemic
Ibuprofen	1.7	4/26	3.18	7.70	3.79	Anti-inflammatory
Meprobamate	12	1/26	21.6	21.6	0.98	Anxiolytic, sedative
Naproxen	2.4	2/26	2.93	4.19	3.10	Analgesic, anti-inflammatory, antipyretic
Pentoxifylline	0.25	1/26	0.46	0.46	0.56	Antidote, vasodilator
Ranitidine	0.6	3/26	1.04	5.60	0.29	Antiulcer, diuretic
Salicylic acid	7.5	21/26	66.4	146	2.24	Analgesic, anti-inflammatory, antipyretic, catalytic
Sildenafil	0.10	1/26	0.27	0.27	2.30	Vasoactive agent
Warfarin	7.9	5/26	57.6	171	2.23	Anticoagulant, pesticide, rodenticide
Antibacterials						
Cloxacillin	0.35	1/26	0.56	0.56	3.22	Antibacterial, antibiotic
Lincomycin	0.9	1/26	1.07	1.07	0.29	Antibacterial, antibiotic
Penicillin V	0.33	5/26	1.70	3.10	1.87	Antibacterial, antibiotic
Sulfachloropyridazine	4.6	2/26	3.78	6.40	0.31	Antibacterial, antibiotic, anti-infective
Sulfathiazole	33	3/26	55.8	102	0.72	Antibacterial, antibiotic
Triclocarban	0.02	3/26	4.71	8.99	4.90	Antibacterial, anti-infective, disinfectant
Triclosan	3.6	3/26	71.3	87.5	4.66	Antibacterial, antiseptic, disinfectant
Endocrine disrupting						
Bisphenol A	146	3/26	397	3576	3.64	Fungicide, pesticide, plasticizer
Caffeine	14	6/26	68.3	8692	0.16	Central nervous system stimulant
Testosterone	2.6	5/26	4.65	12.2	3.27	Androgen
Other						
DEET	5.5	4/26	37.2	63.4	2.26	Insect repellant, pesticide
Thiabendazole	0.11	13/26	0.26	3.92	2.00	Antihelminthic
TCPP	1.1	2/26	53.8	54.4	2.89	Flame retardant
PBDEs (ΣPBDEs 28, 47, 99, 100, 153, 154)	1–2	22/22	83.5	8850	Variable: 5.94–7.82 for BDE congeners analyzed	Flame retardant

Bat species: *M. lucifugus*, *M. sodalis*, *M. septentrionalis*, *and E. fuscus.* Log k_ow_ is KOWWIN v. 1.67 estimated from Estimation Programs Interface software and presented at www.chemspider.com, except for PBDE congeners 28, 47, 99, 100, 153, and 154 KOWWIN from ATSDR (2004). Use is from www.chemspider.com or http://pubchem.ncbi.nlm.nih.gov

*LOD* limit of detection

We removed the wings from bats before shipping them to TestAmerica because these tissues are difficult to homogenize and may increase analytical interference. To achieve the needed sample mass, some individual bat samples shipped to TestAmerica consisted of one to five bats of the same species from the same location and date of collection (15 MYLU, 10 EPFU, and 1 MYSO). Bats analyzed for PBDEs were all individual bats (17 MYLU, 4 EPFU, and 1 MYSE). Bats sent to us from the NYSDOH had their brains removed for rabies testing. All NYSDOH bats were only analyzed for PBDEs.

### Laboratory Analysis: PBDEs

Bat tissues were mechanically homogenized and mixed with hydromatrix sorbent followed by extraction with a pesticide residue quality accelerated solvent extractor 22-ml cell with a 2-cm glass fiber filter in the bottom cell cap in accordance with USEPA Method 3545. Extracts were concentrated to approximately 20 ml by Turbovap. Samples were dissolved in petroleum ether and transferred to a 300-ml glass chromatographic column (no. 420280-0242; Kontes) containing 20 g of Florisil topped with 1 cm of sodium sulfate. The column was eluted with 200 ml 6 % diethyl ether/94 % petroleum ether for fraction 1. Fraction 1 was concentrated to 5 ml and transferred to a silicic acid chromatographic column for additional clean-up to minimize contributions from chemical interferences. Sample extracts were analyzed for six PBDE congeners—BDE nos. 28, 47, 99, 100, 153, and 154—by capillary column, gas chromatography (GC) (Varian 3400 GC with a Varian Stat Data System and a Varian 8200 Autosampler), with electron capture detection.

### Laboratory Analysis: PPCPs

The analytical procedure used for this study determined 75 PPCP target compounds and included four stages: sample compositing and homogenization, sample extraction and clean-up, high-performance liquid chromatography (HPLC)–tandem mass spectrometry (LC/MS/MS) analysis, and data reduction (summarized later in the text and detailed in the Supplementary Material_Methods). PPCP target analytes are listed in Table S1.

One or more bat carcasses with nominal total mass of 4 gm were composited and cryogenically homogenized to create tissue mass for subsequent extraction and analysis of each bat composite. Each 4-g sample homogenate, corresponding to a single bat composite or quality control (QC) aliquot, was split into two equal aliquots (nominal 2-g mass) for separate acid- and base-buffered extraction procedures. QC aliquots included reagent/method blanks, spiked reagent blanks, sample duplicates, and spiked sample duplicates. Each 2-g sample and QC tissue aliquot was added to either 10 ml of phosphate buffer solution (0.14 M monobasic sodium phosphate and 8.5 % phosphoric acid) or ammonium hydroxide solution (0.2 M) and spiked with 46 isotopically labeled analogs of the PPCP analytes. Twenty milliliters of acetonitrile (HPLC grade) was added to each sample, and the tissue/solvent mixture was placed in a sonication bath for 30 min. After sonication, the buffer/acetonitrile solution was decanted, and the tissue was subjected to microwave-assisted extraction (MAE; Milestone Ethos) with 20 ml of 1:1 solution of methanol (pesticide grade) and acetone (HPLC grade), then decanted and followed by another MAE extraction with 20 ml of 1:1 solution of toluene (reagent grade) and acetone. The extracts were then concentrated and solvent-exchanged to water. The aqueous extract was pH-adjusted and added to a hydrophilic–lipophilic balance clean-up column (6 cc/500 mg; Waters Oasis HLB), then eluted with 8 ml of methanol (for acid-buffered extracts) or 8 ml of 2 % formic acid/methanol (for base-buffered extracts) followed by 6 ml of 1:1 solution of methanol and acetone. After clean-up, the resulting methanolic extracts were split and concentrated to 1 ml of methanol and 1 ml of in a 9:1 solution of water and methanol for subsequent analysis.

The instrumental analysis was performed using a Waters Quattro LC/MS/MS operated in positive and negative electrospray mode while monitoring characteristic precursor–product mass transitions for each target analyte with quantitation by the isotope-dilution or internal standard technique. These analytical procedures were performed largely as described in USEPA Method 1694 (USEPA 2007) for the determination of PPCPs in a variety of matrices. Minor modifications to the analytical and data processing procedures in Method 1694 were applied to accommodate target analytes not included in the reference method and to reduce the potential for false-positive results due to matrix interferences encountered in the bat tissue matrix. These modifications are described in detail in the Supplementary Material_Methods.

### Data Analysis

Twenty-four of 75 PPCPs passed quality assurance/quality control (QA/QC) screening with recoveries and method performance deemed acceptable. For PPCPs, data passed QA/QC screening if the detected concentration exceeded 5× the mean concentration in the method blanks, if matrix spike recoveries fell within the USEPA Method 1694 guidelines and relative percent difference in matrix spike recoveries were <50 % (Table S1). If no USEPA matrix spike recovery guidelines were available, data were rejected if matrix spike recoveries fell outside of a 70 to 150 % range. Twenty-three of 75 PPCPs analytes had no detections in any sample. Of the 24/75 PPCP analytes that passed QA/QC screening and were detected, 15 analytes were detected in ≤3 samples. PBDEs were detected in all bat samples with recoveries and method performance deemed acceptable for all data. Only salicylic acid and PBDEs passed QA/QC screening and were also detected in nearly all samples. Respective detection frequencies were 21 of 26 analytes for salicylic acid and 22 of 22 analytes for PBDEs.

All statistical analyses were performed using SAS 9.3 for Windows using detected concentrations only. Individual samples were comprised of one to five bat carcasses. For emerging contaminants, all composite bat samples consisted of bats of the same species collected on the same date at the same location. PBDE analyses were performed in a different set of bats with only one bat per sample.

We explored seasonality in salicylic acid concentration by testing for a difference between bats collected in April to August (*n* = 13) compared with bats collected in February to March (*n* = 6). Nonparametric analysis was indicated because neither untransformed nor log_10_-transformed salicylic concentrations were confirmed to be normally distributed in the small sample of winter-collected bats (Shapiro–Wilk; *n* = 6). We applied Wilcoxon rank sum exact test to check for a statistically significant difference in salicylic acid concentration based on a comparison of mean rank scores between summer and winter groups.

Seasonality of PBDE concentration was also evaluated between bats collected in April to August (*n* = 11) and bats collected in February to March (*n* = 8). Shapiro–Wilk test using log_10_-transformed concentration data confirmed normal distribution of total PBDEs in both summer-collected (*p* = 0.27) and winter-collected (*p* = 0.96) bats. Because the variance differed between the two groups (folded *F* test for equality of variance; *p* = 0.02), the Satterthwaite method for conducting *t* test with unequal variances was used to check for a difference in mean log_10_-transformed total PBDEs between seasons.

Nondetects were excluded from statistical analyses because sample limits of detection varied between samples due to significant variation in chromatographic noise *versus* internal standard response. For certain compounds, very high sample detection limits associated with nondetects resulted in surrogate values (*e.g.*, ½ the detection limit) that exceeded some detected concentrations.

## Results

**Fig. 1:**
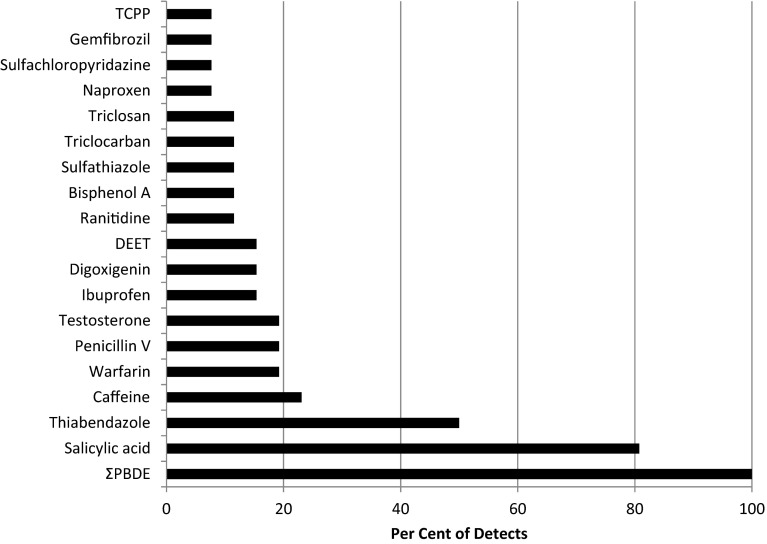
Percent detection of contaminants of emerging concern (CECs) in bat carcasses from the northeastern United States (based on 22 bat carcasses for ΣPBDE and 26 bat carcass samples for remaining CECs). Bat species—*Myotis lucifugus*, *M. sodalis*, *M. septentrionalis*, *Eptesicus fuscus*

All mean CEC concentrations are reported as geometric mean on a wet-weight basis (Table 1). Geometric means are used as the metric of central tendency due to skewed sample data distributions that would, for ΣPBDE, bisphenol A, and caffeine, have yielded arithmetic means disproportionately influenced by a few high values. PBDEs were detected in 100 % (22 of 22) of bat carcasses (Fig. 1). Of the remaining CECs, the most frequently detected in the bat samples were salicylic acid (81 %), thiabendazole (50 %), caffeine (23 %), warfarin (19 %), and penicillin V (19 %) (Fig. 1). CECs detected in 11–15 % of bat composite samples were digoxigenin, ibuprofen, ranitidine, sulfathiazole, triclocarban, triclosan, bisphenol A, and DEET. CECs detected in 4–8 % of bat samples were diltiazem, gemfibrozil, meprobamate, naproxen, pentoxyfylline, sildenafil, cloxacillin, lincomycin, sulfachloropyridazine, and tris(1-chloro-2-propyl) phosphate (TCPP) (Fig. 1, Table 1)

**Fig. 2:**
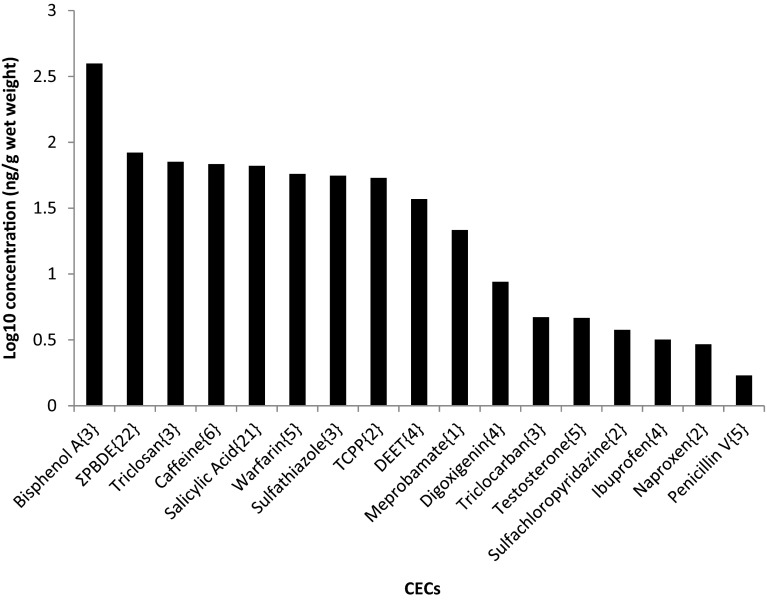
Geometric mean concentration of contaminants of emerging concern (CECs) in bat carcasses from the northeastern United States. Geometric means are calculated from detected concentrations only and do not include non-detects. The number of detects for a given compound is shown in {} after the compound name on the x-axis. Bat species—*Myotis lucifugus*, *M. sodalis*, *M. septentrionalis*, *Eptesicus fuscus*

**Fig. 3:**
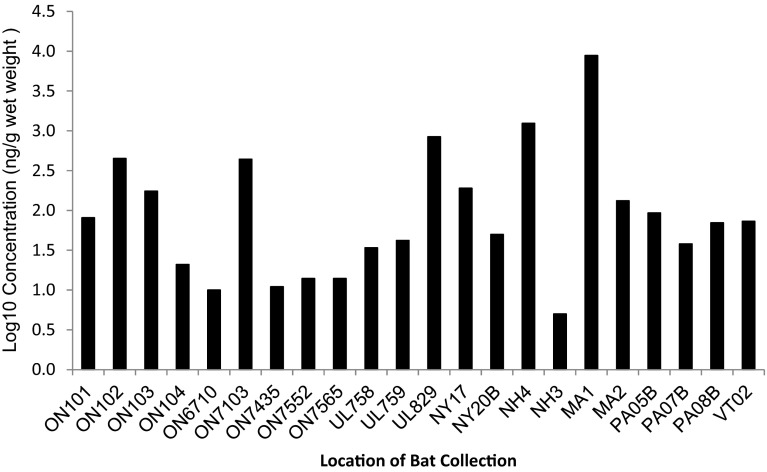
Total polybrominated diphenyl ethers (PBDE) in bat carcasses from the northeastern United States. Total PBDE is calculated as the sum of BDE 28, BDE 47, BDE 99, BDE 100, BDE 153 and BDE 154. Bats with the prefix ON are from Onondaga County, NY; UL are from Ulster County, NY; NY are from New York State; NH are from New Hampshire; MA are from Massachusetts; PA are from Pennsylvania; VT is from Vermont. Bat species—*Myotis lucifugus*, *M. sodalis*, *M. septentrionalis*, *Eptesicus fuscus*

CECs present at the highest geometric mean wet-weight concentrations in bat carcasses were bisphenol A (397 ng/g), ΣPDBE congeners 28, 47, 99, 100, 153, and 154 (83.5 ng/g), triclosan (71.3 n/g), caffeine (68.3 ng/g), salicylic acid (66.4 ng/g), warfarin (57.6 ng/g), sulfathiazole (55.8 ng/g), TCPP (53.8 ng/g), and DEET (37.2 ng/g) (Fig. 2). ΣPDBEs in bat samples ranged from a low concentration of 5 ng/g in a bat from New Hampshire to a high concentration of 8850 ng/g in a bat from Massachusetts (Fig. 3).

There was no significant difference in the concentration of ΣPDBE between summer (April to August) and winter-collected (September to March) bats (*p* = 0.85). For salicylic acid, the concentration for summer-collected bat samples (79.8 ng/g) was significantly greater than that for winter-collected bat samples (46.0 ng/g) (*p* = 0.009).

## Discussion

This study was intended to provide preliminary data on whether CECs are present in bat tissues, interpret observed body burdens in terms of potential health effects, and propose recommendations on whether CECs should be given additional scrutiny for potential impacts to bats. This study documents the presence of CECs in bats and considers the implications of CECs for the health of bats.

North American bats are experiencing population level losses due to WNS, a fungal disease that has its greatest impacts during hibernation and emergence (Reeder et al. 2012; Verant et al. 2014). Bats in torpor experience reduced immune function, thus potentially compromising their ability to combat WNS (Bouma et al. 2010; Meteyer et al. 2012). In addition, fat reserves are metabolized during hibernation (Geluso et al. 1976; Clark and Shore 2001), a process that can mobilize contaminants to the brain and other tissues coincident with reduced immune function. CECs, such as PBDEs, bisphenol A, and triclosan, may further diminish immune competence as has been shown in other mammals (Thuvander and Darnerud 1999; Martin et al. 2007; Liu et al. 2012; Vandenberg et al. 2013). A number of the CECs detected in bats are known to affect hibernation (Jinka et al. 2011) or influence prostaglandin synthesis (Vane 1971; Bjorkman 1998; Xu et al. 1999) or thyroid balance (Zhou et al. 2001; Hallgren et al. 2001; Zhang et al. 2009; Zorilla et al. 2009; Kodavanti et al. 2010), thus potentially impacting hibernation (Takahata et al. 1996; Prendergast et al. 2002; Arnold et al. 2012). In addition, there is emerging evidence that bacteria may have the potential to act as protective probiotics in the defense of *P. destructans* (Cornelison et al. 2014; Hoyt et al. 2015); hence, antibacterials in the environment may affect beneficial bacteria in bats. The implications of specific CECs for bat health and conservation are discussed further in the text.

### Antibacterial–Fungal Relationships and WNS

**Fig. 4:**
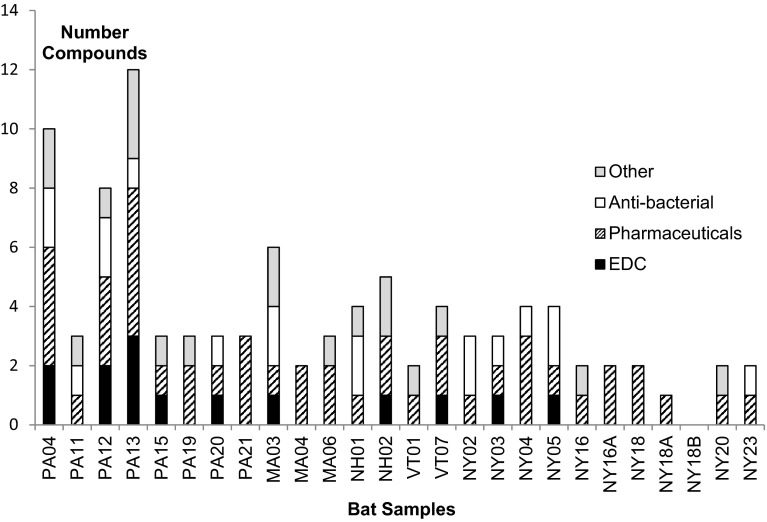
Number of non-PBDE detected contaminants of emerging concern (CECs) in bat carcass samples from the northeastern United States. PA bats are from Pennsylvania; MA bats are from Massachusetts; NH bats are from New Hampshire; VT bats are from Vermont; NY bats are from New York. Bat species—*Myotis lucifugus*, *M. sodalis*, *M. septentrionalis*, *Eptesicus fuscus. EDC endocrine disrupting compounds*

Twelve of 26 bat composite samples contained at least 1 antibiotic/antibacterial compound (Fig. 4). The detection of these compounds in some of the bat samples is of scientific interest because the fungal–bacterial relationship may be relevant to WNS—a disease caused by the fungal pathogen *P. destructans*. An antagonistic relationship can occur between bacteria and fungi with bacteria competing with fungi or inhibiting fungal growth (Mille-Lindbloom et al. 2006; Brucker et al. 2008; Loudon et al. 2014). Specific to the possible relationship between bacteria and *P. destructans* in bats, a natural antagonism was shown between *P. destructans* and the bacteria *Rhodococcus rhodochrous* (Cornelison et al. 2014). Researchers found that *Rhodococcus* completely and permanently inhibited spore germination of *P. destructans* and slowed the growth of the fungal hyphae. In subsequent work by Hoyt et al. (2015), 6 *Pseudomonas* bacteria isolates cultured from the skin of bats suppressed the growth of *P. destructans* in vitro. Both Cornelison et al. (2014) and Hoyt et al. (2015) recommended that bacteria be further studied as probiotics to protect bats from WNS. We found no evidence in the literature linking the environmental occurrence of antibiotics and antibacterials with the proliferation of wildlife fungal disease. However, considering the extreme mortality caused by wildlife fungal diseases such as WNS in bats and chytrid fungus (*Batrachochytrium dendrobatidis*) in amphibians (Daszak et al. 1999), this interaction warrants further scientific study.

### Potential CEC Influence on Hibernation

One of the clinical signs of WNS is frequent arousals from hibernation that contribute to WNS-associated mortality (Reeder et al. 2012). A number of the CECs detected in bats (caffeine, salicylic acid, ibuprofen, naproxen, PBDEs, and triclosan) may influence hibernation and emergence from hibernation.

Caffeine is an antagonist of adenosine, a compound that has been found to induce hibernation in arctic ground squirrels (*Urocitellus parryii*). Exposure of arctic ground squirrels to the adenosine antagonist, cyclopentyltheophylline, reversed torpor onset in this species (Jinka et al. 2011). Caffeine was detected in 23 % of bat samples from this study (geometric mean 68.3 ng/g) with one *M. lucifugus* from NY collected as part of the NYSDOH surveillance program having 8692 ng/g caffeine. Caffeine is frequently detected in surface water and is considered an indicator of urban pollution (Ferreira 2005). Caffeine has an extremely low log K_ow_ (0.16) (Table 1) and a short half-life of <3 h in mice (Burg and Werner 1972) and <3.5 h in humans (Axelrod and Reichenthal 1953). Its detection in bat tissues from this study suggests a recent exposure of some bats to caffeine.

Salicylic acid and other NSAIDS reduce inflammation by inhibiting the synthesis of prostaglandins (Vane 1971; Bjorkman 1998; Xu et al. 1999), compounds that are believed to play a role in hibernation (Takahata et al. 1996; Prendergast et al. 2002; Arnold et al. 2012). Alpine marmots (*Marmota marmota*) and yellow-bellied marmots (*M. flaviventris*) experienced decreased prostaglandin PGE_2_ and increased prostaglandin PGD_2_ in the brain during hibernation (Arnold et al. 2012). In Asian chipmunks (*Tamias asiaticus*), prostaglandin PGD_2_ increased in the brain to a maximum in February and declined by April suggesting a correlation between PGD_2_ and hibernation (Takahata et al. 1996). Infusion of prostaglandin PGE_2_ into the brains of hibernating golden-mantled ground squirrels (*Spermophilus lateralis*) caused periodic arousals along with increased body temperature (Prendergast et al. 2002). These studies suggest that prostaglandin levels are related to hibernation in at least these three mammalian species. Twenty-one of 26 bats had salicylic acid in their tissues, four bats had ibuprofen, and two bats had naproxen. Summer-collected bats had significantly higher detected salicylic acid concentrations than winter bats (*p* = 0.009).

PBDEs, by way of their potential to alter thyroid homeostasis (Zhou et al. 2001; Hallgren et al. 2001; Zhang et al. 2009), may influence hibernation or emergence from hibernation in bats. Thyroid function in hibernating mammals changes seasonally; the thyroid is generally most active in spring when animals are emerging from hibernation and least active when they are entering or in hibernation (Kayser 1961). Little brown bats (*M. lucifugus*) exhibited peak plasma thyroxine (T4) in May at emergence from hibernation and the lowest concentration of plasma T4 in September (Damassa et al. 1995). In *M. lucifugus* and *Pipestrillus pipestrillus*, the emergence from hibernation was found to be associated with structural changes to the thyroid (Nunez et al. 1974).

Thyroid function in mammals has been shown to be influenced by PBDEs. Weanling rats dosed with three commercial PBDE mixtures exhibited dose-dependent decreases of serum total T4 (Zhou et al. 2001). Both rats and mice dosed with a PBDE mixture or DE-47 exhibited decreased plasma free and total T4 (Hallgren et al. 2001). Juvenile female ranch mink (*Mustela vison*) dosed with a PBDE mixture exhibited increased T4; mink in all age and sex groups experienced reduced triiodothyronine (T3) in a PBDE dose-dependent manner (Zhang et al. 2009). Pregnant rats dosed with a PBDE mixture experienced reduced serum T4 at the two highest doses (10.2 and 30.6 mg/kg/day) (Kodavanti et al. 2010). The offspring of the dosed females had significantly lower serum T4 until weaning. Male offspring of the high-dose (30.6 mg/kg/day) dams on postnatal day 22 had wet-weight PBDE concentrations in blood, frontal cortex, and liver of approximately 1, 8, and 50 µg/g, respectively (from Fig. 12 of Kodavanti et al. 2010). This compares with wet-weight ΣPBDEs in whole bats from this study of 0.005–8.85 µg/g (geometric mean 0.083 µg/g). Although we are unable to make direct comparisons between our bat carcass data and the rat tissue data from Kodavanti et al. (2010), at least one bat carcass from our study had a ΣPBDE concentration (8.85 µg/g) that is the same order of magnitude as PBDE tissue (blood, brain, and liver) concentrations in rats exhibiting reduced T4 in response to PBDE dosing (1–50 µg/g).

Triclosan has also been found to decrease serum T4 in male rats in a dose-dependent manner at doses of ≥30 mg/kg/day (Zorrilla et al. 2009). We detected triclosan in 3 of 26 bats with at a geometric mean concentration of 71.3 ng/g.

Caffeine, NSAIDs, PBDEs, and triclosan may have the potential in bats to alter the concentrations of hormones such as T4 and T3 or hormone-like substances such as adenosine and prostaglandins (Shimizu and Nakamura 1985; Zentella de Piña et al. 1989), all of which may play a role in bat hibernation or emergence. Further evaluation of these CECs in bats, with a focus on seasonal changes in tissue concentration in association with hibernation, should be considered.

### CECs and Immune Function

Bats experience reduced immune function during torpor (Meteyer et al. 2012). CECs detected in this study (PBDEs, bisphenol A, and triclosan) may exert immunotoxicity. Alterations in immune function have been reported in rats, mice, rats, and ranch mink dosed with PBDE congeners or PBDE mixtures (Thuvander and Darnerud 1999; Martin et al. 2007; Liu et al. 2012). Bisphenol A alters immune function in rodents and humans (Vandenberg et al. 2013). Triclosan has been correlated with negative effects on human immune function (Clayton et al. 2011). There is the potential for CECs to further affect the already reduced immune function of hibernating bats.

### NSAIDs and Systemic Effects

Pharmaceutical compounds are designed to have biological effects at low concentrations (Arnold et al. 2013). They are also generally designed for their efficacy at addressing ailments/symptoms in mammals, particularly humans and our mammalian livestock and pets. It is possible that bats may be equally susceptible to the effects of these chemicals that have been specifically designed to influence mammalian systems.

We previously discussed the potential effect of NSAIDs on hibernation. The NSAIDs diclofenac and ibuprofen have been detected in the hair of Eurasian otters (*Lutra lutra*) from six counties in England. Trophic transfer is a likely route of exposure between these two drug residues, which are discharged into waterways by humans, and this high trophic-level, semiaquatic wild mammal (Richards et al. 2011). Renal lesions were observed in approximately 10 % of Eurasian otters studied as part of that same otter health study (Simpson et al. 2011). The investigators hypothesized a link between the two NSAIDs and the identified lesions. Diclofenac and its metabolite, 4-hydroxydiclofenac, were analyzed but not detected in bats from this study. However, the NSAIDs ibuprofen, naproxen, and salicylic acid were detected in bat carcass samples. Considering our results and the large body of work linking diclofenac with renal failure and mass mortality of Asian vultures (*Gyps spp.*) (Oaks et al. 2004), a more critical evaluation of NSAIDs in bats is warranted.

The presence of CECs in bats reveals the direct connection between chemicals that humans and livestock use and bats that share our ecosystem. Effective doses or concentrations of these CECs have not been measured directly in bats. Nevertheless, studies in surrogate mammalian species indicate that CECs—such as antibacterials, caffeine, salicylic acid, NSAIDs, bisphenol A, triclosan and PBDEs—have the potential to affect bacterial–fungal balance, regulation of torpor, immune function, or other physiological systems in bats. We suggest that further research is warranted to investigate exposure–response relationships of some of the CECs in bats or bat surrogates.

## Electronic supplementary material

Supplementary material 1 (DOCX 45 kb)

Supplementary material 2 (DOCX 27 kb)
