# Supplementary material for: Contaminants of Emerging Concern in Bats from the Northeastern United States
Source: Arch Environ Contam Toxicol. 2015 Aug 6;69(4):411–21. doi: 10.1007/s00244-015-0196-x (PMC4600474; doi:10.1007/s00244-015-0196-x)
Supplement: Supplementary file 1 — Supplementary material 1 (DOCX 45 kb) [file 244_2015_196_MOESM1_ESM.docx]

**Contaminants of Emerging Concern in Bats from the Northeastern United States**

Anne L. Secord^1^, Kathleen A. Patnode^2^, Charles Carter^3^, Eric Redman^4^, Daniel J. Gefell^1^, Andrew R. Major^5,^ Daniel W. Sparks^6^

(1) U.S. Fish and Wildlife Service, 3817 Luker Road,

Cortland, New York 13045; 607-753-9334; [Anne_secord@fws.gov](mailto:Anne_secord@fws.gov)

(2) U.S. Fish and Wildlife Service, 110 Radnor Road,

Suite 101, State College, PA 16801

(3) TestAmerica, 3275 S. Tioga Way, Las Vegas, NV 89117

(4) TestAmerica, 880 Riverside Parkway, West Sacramento, CA 95605

(5) U.S. Fish and Wildlife Service, 70 Commercial St., Suite 300,

Concord, NH 03301

(6) U.S. Fish and Wildlife Service, 620 S. Walker St., Bloomington, IN 47403

**Supplementary material - detailed laboratory analysis for contaminants of emerging concern – pharmaceuticals and personal care products**

The instrumental analysis was conducted using a Waters Quattro LC/MS/MS operated in positive and negative electrospray mode while monitoring characteristic precursor-product mass transitions for each target analyte, with quantitation by the isotope-dilution or internal standard techniques. These analytical procedures were performed largely as described in USEPA Method 1694 for the determination of pharmaceuticals and personal care products in a variety of matrices. As previously noted, each 4 gram bat sample homogenate corresponding to a single bat sample or QC aliquot was split into 2 equal aliquots (nominally 2 grams each) for sequential extraction via separate acid-buffered and base-buffered extraction procedures. The extract from each 2 gram sample aliquot was concentrated to a 1 ml methanol extract and a 1 ml aqueous (9:1 water/methanol) extract. Thus, each 4 gram sample aliquot generated 4 individual extracts for analysis (acid/methanol, acid/aqueous, base/methanol, and base/aqueous), wherein the extraction and analysis conditions were optimized for a subset of the 75 targeted emerging contaminants. Each of the 4 extracts was analyzed for all 75 analytes, and QC data were evaluated to select the most reliable single result for each analyte from the 4 analyses.

Aqueous extracts were analyzed using optimized HPLC and MS conditions as outlined in Tables 1 - 4 below:

| Table 1. HPLC Configuration | |
| --- | --- |
| **Parameter** | **Configuration** |
| Column | C18- 100 x 2.1mm, 3.5um particle size (Waters XTerra C18 MS (PN 186000404) |
| Column Temperature | 40 °C |
| Injection Volume | 25 uL loop with partial overflow (injection volume is consistent with the volume used for the initial calibration). |

###

| Table 2. LC Solvent System | |
| --- | --- |
| **Reservoir** | **Solvent** |
| A | 0.1% acetic acid and 0.1% ammonium acetate in water |
| B | 1:1 methanol:acetonitrile |
| C | Not Used |
| D | Not Used |

| Table 3. LC Gradient Program | | | | |
| --- | --- | --- | --- | --- |
| **Time**  **(minutes)** | **A**  **%** | **B**  **%** | **Flow Rate**  **mL/min** | **Curve** |
| 0.00 | 95 | 5 | 0.20 | Initial |
| 1.00 | 95 | 5 | 0.20 | 6 |
| 25.00 | 0 | 100 | 0.20 | 6 |
| 27.00 | 0 | 100 | 0.20 | 6 |
| 27.10 | 95 | 5 | 0.20 | End of Run |

| Table 4. Tandem MS conditions | | | |
| --- | --- | --- | --- |
| Capillary Voltage | 2.95 kV | Desolvation Gas Flow | 500 L/hr |
| Cone Voltage | Refer to Table 9 | Collision Energy | Refer to Table 9 |
| Extractor Voltage | 4.00 V | Multiplier Voltage | 750 V |
| RF lens | 0.3 V | Collision Gas Flow | 0.35 mL/min |
| Source Temperature | 140 °C. | Pressure | ~3E-3 mbar |
| Desolvation Temperature | 380 °C. | Scanning Conditions | Refer to Table 9 |
| Cone Gas Flow | 20 L/hr | Dwell/Delay Times (Both Ions) | 0.20 sec / 0.05 sec |

Methanol extracts were analyzed using optimized HPLC and MS conditions as outlined in Tables 5 - 8 below:

| Table 5. HPLC Configuration: | |
| --- | --- |
| **Parameter** | **Configuration** |
| C8 Column | 100 x 2.0 mm, 3um particle size (Phenomenex Luna C8 (PN 00D-4248-B0), or equivalent) |
| Column Temperature | 40 °C |
| Injection Volume | 20 uL loop with partial overflow (injection volume is consistent with the volume used for the initial calibration). |

| Table 6. LC Solvent System | |
| --- | --- |
| **Reservoir** | **Solvent** |
| A | 0.1% ammonium hydroxide in water |
| B | 1:1 methanol:acetonitrile |
| C | Not used |
| D | Not used |

| Table 7. LC Gradient Program | | | | |
| --- | --- | --- | --- | --- |
| **Time**  **(minutes)** | **A**  **%** | **B**  **%** | **Flow Rate**  **mL/min** | **Curve** |
| 0.00 | 90 | 10 | 0.20 | Initial |
| 0.50 | 90 | 10 | 0.20 | 6 |
| 2.00 | 50 | 50 | 0.20 | 6 |
| 10.00 | 0 | 100 | 0.20 | 6 |
| 12.00 | 0 | 100 | 0.20 | 6 |
| 12.50 | 90 | 10 | 0.20 | 6 |
| 17.00 | 90 | 10 | 0.20 | End of Run |

| Table 8. Tandem MS conditions | | | |
| --- | --- | --- | --- |
| Capillary Voltage | 2.95 kV | Desolvation Gas Flow | 500 L/hr |
| Cone Voltage | Refer to Table 9 | Collision Energy | Refer to Table 9 |
| Extractor Voltage | 4.00 V | Multiplier Voltage | 750 V |
| RF lens | 0.3 V | Collision Gas Flow | 0.25 mL/min |
| Source Temp | 130 °C. | Pressure | ~3E-3 mbar |
| Desolvation Temp | 350 °C. | Scanning Conditions | Refer to Table 9 |
| Cone Gas Flow | 50 L/hr | Dwell/Delay Times (Both Ions) | 0.20 sec / 0.05 sec |

| Table 9 | | | | | | | |
| --- | --- | --- | --- | --- | --- | --- | --- |
| MS Conditions and Grouping | | | | | | | |
| Pharmaceuticals, Personal Care Products | | | | | | | |
| **Analyte** | **ESI Mode** | **Transition Precursor>Product** | **Dwell**  **(Sec)** | **Cone Voltage** | **Collision Voltage** | **Delay**  **(sec)** | **Group** |
| Acetaminophen | pos | 152.00 > 110.10 | 0.02 | 30 | 17 | 0.06 | 1 |
| Albuterol | pos | 240.3 >148.2 | 0.02 | 15 | 18 | 0.01 | 5 |
| Atenolol | pos | 267.30 > 145.20 | 0.02 | 35 | 24 | 0.01 | 1,3 |
| Atorvastatin (Lipitor) | neg | 557.50 > 397.50 | 0.02 | 38 | 28 | 0.01 | 2,4 |
| Atrazine | pos | 216.2 > 174 | 0.002 | 18 | 18 | 0.01 |  |
| Azithromycin | pos | 749.10 > 591.10 | 0.02 | 60 | 32 | 0.01 | 1,3 |
| Bisphenol A | neg | 227.2 > 212.20 | 0.02 | 36 | 20 | 0.01 | 2,4 |
| Caffeine | pos | 195.00 > 138.00 | 0.02 | 30 | 20 | 0.01 | 1 |
| Carbadox | pos | 263.1 > 231.1 | 0.02 | 30 | 14 | 0.01 | 1 |
| Carbamazepine | pos | 237.00 > 194.00 | 0.02 | 28 | 20 | 0.01 | 1,3,5 |
| Cimetidine | pos | 253.40 > 158.90 | 0.02 | 23 | 15 | 0.01 | 1,3,5 |
| Clarithromycin | pos | 748.6 > 158.2 | 0.02 | 25 | 30 | 0.01 | 1,3 |
| Cloxacillin | pos | 468 > 160.1 | 0.02 | 25 | 19 | 0.01 |  |
| Cotinine | pos | 177.00 > 79.80 | 0.02 | 27 | 23 | 0.01 | 1,3,5 |
| Codeine | pos | 300.2 > 215.2 | 0.02 | 38 | 24 | 0.01 | 1 |
| Diclofenac | neg |  |  |  |  |  |  |
| N,N-Diethyl-3-methylbenzamide (DEET) | pos | 192.20 > 192.20 | 0.02 | 32 | 7 | 0.01 | 1,3 |
| Digoxigenin | pos | 391.5 > 355.2 | 0.02 | 25 | 16 | 0.01 | 1,3 |
| Digoxin | pos | 798.50 > 97.20 | 0.02 | 20 | 28 | 0.01 | 1,3 |
| Diltiazem | pos | 415.20 > 177.90 | 0.02 | 28 | 26 | 0.01 | 1,3 |
| 1,7-Dimethylxanthine | pos | 181.1 > 124.1 | 0.02 | 36 | 21 | 0.01 | 1 |
| Diphenhydramine hydrochloride (Benadryl or Dimedrol) | pos | 256.30 > 167.20 | 0.02 | 16 | 18 | 0.01 | 1,3 |
| Equilin | neg | 267 > 265  267 > 143 | 0.02 | 40  40 | 24  38 | 0.01 |  |
| Equilenin | neg | 265.1>221.1 | 0.02 | 55 | 36 | 0.01 | 7 |
| Estriol | neg | 287.1>145 | 0.02 | 65 | 45 | 0.01 | 7 |
| Estrone | neg | 269.0>145.0 | 0.02 | 65 | 40 | 0.01 | 7 |
| Erythromycin A | pos | 734.80 > 158.30 | 0.02 | 35 | 28 | 0.01 | 1,3 |
| Flumequine | pos | 262.20 > 202.00 | 0.02 | 29 | 38 | 0.01 | 1,3 |
| Fluoxetine | pos | 310.10 > 148.10 | 0.02 | 17 | 8 | 0.01 | 3 |
| Gemfibrozil | neg | 249.00 > 120.70 | 0.02 | 16 | 15 | 0.01 | 2,4 |
| Hydrocodone | pos | 300.1 > 199.2 | 0.02 | 24 | 30 | 0.01 |  |
| 4’-Hydroxydiclofenac | neg | 310 > 166  310 > 266 | 0.02 | 20  20 | 28  14 | 0.01 |  |
| Ibuprofen | neg | 205.00 > 161.00 | 0.02 | 18 | 7 | 0.01 | 2,4 |
| Iopromide | pos | 791.20 > 572.20 | 0.02 | 38 | 27 | 0.01 | 1 |
| Lincomycin | pos | 407.10 > 126.00 | 0.02 | 40 | 30 | 0.01 | 3,5 |
| Lorazepam | pos | 321.20 > 275.30 | 0.02 | 35 | 17 | 0.01 | 1,3 |
| Meprobamate | pos | 219.1 > 158 | 0.02 | 24 | 10 | 0.01 |  |
| Methadone | pos | 310.40 > 265.40 | 0.02 | 26 | 16 | 0.01 | 1,3 |
| Miconazole nitrate | pos | 417 > 161.1 | 0.02 | 36 | 33 | 0.01 |  |
| Morphine | pos | 286.30 > 165.30 | 0.02 | 50 | 50 | 0.01 | 1,3 |
| Naproxen | neg | 229.10 > 169.90 | 0.02 | 17 | 15 | 0.01 | 2,4 |
| Nifedipine | pos | 347.2 > 315.2 | 0.02 | 15 | 10 | 0.01 | 1,3 |
| Ormetoprim | pos | 275.20 > 259.10 | 0.02 | 37 | 29 | 0.01 | 1 |
| Oxolinic acid | pos | 262 >244.1 | 0.02 | 28 | 19 | 0.01 |  |
| Oxybenzone | pos | 229.1 > 151.0 | 0.02 | 27 | 20 | 0.01 | 8 |
| Progesterone | pos | 315.2>96.9 | 0.02 | 38 | 25 | 0.01 | 8 |
| Penicillin G | pos | 367.00 > 160.00 | 0.02 | 27 | 16 | 0.01 | 1 |
| Penicillin V | pos | 383.20 > 160.10 | 0.02 | 27 | 16 | 0.01 | 1 |
| Pentoxifyline | pos | 279.2 > 181.1 | 0.02 | 18 | 18 | 0.01 |  |
| Phenytoin sodium (Dilantin or Eptoin) | neg | 251.30 > 102.10 | 0.02 | 35 | 22 | 0.01 | 2 |
| Primidone | pos | 219.30 > 162.20 | 0.02 | 30 | 13 | 0.01 | 1 |
| Ranitidine | pos | 315.10 > 176.00 | 0.02 | 23 | 16 | 0.01 | 1,3,5 |
| Roxithromycin | pos | 837.6 > 679.5 | 0.02 | 40 | 21 | 0.01 |  |
| Salicylic Acid | neg | 137.10 > 93.10 | 0.02 | 26 | 17 | 0.01 | 2 |
| Sildenafil (Viagra) | pos | 475 > 100 | 0.02 | 54 | 26 | 0.01 |  |
| Sucralose | pos | 419 > 221.2 | 0.02 | 24 | 22 | 0.01 |  |
| Sulfachloropyridazine | pos | 285.10 > 156.10 | 0.02 | 26 | 14 | 0.01 | 1 |
| Sulfadiazine | pos | 251.2 > 156 | 0.02 | 25 | 16 | 0.01 | 1 |
| Sulfadimethoxine | pos | 311.30 > 156.00 | 0.02 | 27 | 26 | 0.01 | 1 |
| Sulfamerazine | pos | 265.20 > 156.10 | 0.02 | 26 | 18 | 0.01 | 1 |
| Sulfamethazine | pos | 279.10 > 155.60 | 0.02 | 25 | 20 | 0.01 | 1 |
| Sulfamethizole | pos | 271.10 > 156.10 | 0.02 | 20 | 15 | 0.01 | 1 |
| Sulfamethoxazole | pos | 254.10 > 156.00 | 0.02 | 28 | 18 | 0.01 | 1 |
| Sulfanilamide | pos | 189.90 > 155.80 | 0.02 | 14 | 14 | 0.01 | 1 |
| Sulfathiazole | pos | 256.30 > 156.20 | 0.02 | 25 | 14 | 0.01 | 1 |
| Testosterone | pos | 289.2>96.9 | 0.02 | 40 | 25 | 0.01 | 8 |
| Tris(2-chloroethyl)phosphate (TCEP) | pos | 285 > 161 | 0.02 | 30 | 14 | 0.01 | 1 |
| Tris(1-chloro-2-propyl)phosphate (TCPP) | pos | 327.10 > 99.20 | 0.02 | 18 | 20 | 0.01 | 1 |
| Tris(1,3-dichloro-2-propyl)phosphate (TDCPP) | pos | 430.80 > 99.20 | 0.02 | 18 | 20 | 0.01 | 1,3 |
| Thiabendazole | pos | 202 > 175 | 0.02 | 30 | 26 | 0.01 | 1,3 |
| Triclocarban | neg | 313.00 > 160.00 | 0.02 | 25 | 15 | 0.01 | 4 |
| Triclosan | neg | 287.00 > 287.00 | 0.02 | 16 | 5 | 0.01 | 4 |
| Trimethoprim | pos | 291.10 > 230.00 | 0.02 | 40 | 26 | 0.01 | 3 |
| Tylosin | pos | 916.10 > 173.90 | 0.02 | 45 | 35 | 0.01 | 3 |
| Warfarin | neg | 307.10 > 307.10 | 0.02 | 37 | 6 | 0.01 | 2,4 |
| ***Labeled IDA*** | | | | | | | |
| 13C2-15N-Acetominophen | pos | 155.00 > 110.90 | 0.02 | 25 | 16 | 0.01 | 1 |
| Albuterol-d3 | pos | 243.30 > 225.30 | 0.02 | 13 | 11 | 0.01 | 1 |
| Atenolol-d7 | pos | 274.30 > 79.10 | 0.02 | 35 | 24 | 0.01 | 1 |
| Azithromycin-d3 | pos | 752.6 > 594.4 | 0.02 | 60 | 33 | 0.01 |  |
| Bisphenol A-d6 | pos | 233 > 215 | 0.02 | 36 | 20 | 0.01 |  |
| 13C3-Caffeine | pos | 198.00 > 139.80 | 0.02 | 30 | 20 | 0.01 | 1 |
| Carbamazepine-d10 | pos | 247.00 > 204.00 | 0.02 | 35 | 20 | 0.01 | 1,3,5 |
| Cimetidine-d3 | pos | 256.20 > 162.10 | 0.02 | 26 | 15 | 0.01 | 1,3,5 |
| 13C3-Ciproflaoxacin:HCl | pos | 336.30 > 318.30 | 0.02 | 22 | 22 | 0.01 | 1 |
| Codeine-d6 | pos | 306.3 > 218.4 | 0.02 | 41 | 25 | 0.01 | 1 |
| Cotinine-d3 | pos | 179.90 > 79.80 | 0.02 | 27 | 22 | 0.01 | 1,3,5 |
| Demeclocycline-d3-HCl | pos | TBD | 0.02 | TBD | TBD | 0.01 |  |
| DEET-d6 | pos | 198.30 > 198.30 | 0.02 | 32 | 7 | 0.01 | 1 |
| Diazepam-d5 | pos | 290.20 > 227.30 | 0.02 | 30 | 30 | 0.01 | 1 |
| Diltiazem-d6 | pos | 421.2 > 178 | 0.02 | 28 | 28 | 0.01 |  |
| 13C4, 15N3-1,7-Dimethylxanthine | pos | 187.8 > 129.0 | 0.02 | 34 | 21 | 0.01 |  |
| Diphenhydramine-d5 HCl | pos | 261.30 > 172.10 | 0.02 | 10 | 18 | 0.01 | 1 |
| 13C2-Erythromycin | pos | 736.50 > 160.30 | 0.02 | 35 | 28 | 0.01 | 1 |
| Fluoxetine-d5 | pos | 315.20 > 152.90 | 0.02 | 17 | 8 | 0.01 | 2,4 |
| Gemfibrozil-d6 | neg | 255.10 > 120.80 | 0.02 | 17 | 15 | 0.01 | 2,4 |
| Hydrocodone-d6 | pos | 306.14 > 202 | 0.02 | 24 | 30 | 0.01 |  |
| 13C6-4’-Hydroxy Diclofenac | neg | 316 > 272  318 > 274 | 0.02 | 20 | 12 | 0.01 |  |
| 13C3-Ibuprofen | neg | 208.00 > 163.00 | 0.02 | 17 | 7 | 0.01 | 2,4 |
| Iopromide-d3 | pos | 794.90 > 575.90 | 0.02 | 38 | 25 | 0.01 | 1 |
| Lincomycin-d3 | pos | 410.3 > 129.3 | 0.02 | 39 | 39 | 0.01 |  |
| Lorazepam-d4 | pos | 325.10 > 279.20 | 0.02 | 30 | 16 | 0.01 | 1,3 |
| Meprobamate-d7 | pos | 226.1 > 165 | 0.02 | 24 | 10 | 0.01 |  |
| Minocycline-d6-dihydrochloride | pos | 464 > 447 | 0.02 | 26 | 20 | 0.01 |  |
| 13C-Naproxen-d3 | neg | 233.00 > 168.90 | 0.02 | 18 | 36 | 0.01 | 2,4 |
| 13C6-Oxybenzone | pos | 235 > 151 | 0.02 | 35 | 19 | 0.01 |  |
| Pentoxifyline-d6 | pos | 285.2 > 187.1 | 0.02 | 18 | 18 | 0.01 |  |
| Phenytoin-d10 | neg | 261.20 > 106.20 | 0.02 | 28 | 22 | 0.01 | 2,4 |
| Primidone-d5 | pos | 224.20 > 167.20 | 0.02 | 24 | 12 | 0.01 | 1,3 |
| Salicyclic Acid-d6 | neg | 141.00 > 97.10 | 0.02 | 26 | 18 | 0.01 | 2 |
| Sucralose-d6 | pos | 427 > 427 | 0.02 | 20 | 6 | 0.01 |  |
| 13C6-Sulfamethazine | pos | 285.30 > 162.20 | 0.02 | 25 | 20 | 0.01 | 1 |
| 13C6-Sulfamethoxazole | pos | 260.10 > 161.90 | 0.02 | 28 | 17 | 0.01 | 1 |
| 13C6-Thiabendazole | pos | 208.00 > 181.00 | 0.02 | 30 | 29 | 0.01 | 1,3 |
| 13C12-Triclocarban | neg | 319.00 > 160.00 | 0.02 | 25 | 15 | 0.01 | 4 |
| 13C6Triclosan | neg | 299.00 > 299.00 | 0.02 | 16 | 5 | 0.01 | 4 |
| 13C3-Trimethoprim | pos | 294.10 > 233.10 | 0.02 | 36 | 25 | 0.01 | 3 |
| Warfarin-d5 | neg | 312.40 > 312.40 | 0.02 | 37 | 6 | 0.01 | 2,4 |
| Equilenin-d3 | neg | 268.1>222.2 | 0.02 | 55 | 36 | 0.01 | 8 |
| Estriol-d3 | neg | 290.2 > 147.2 | 0.02 | 65 | 43 | 0.01 | 8 |
| Estrone-d4 | neg | 273.2>147.2 | 0.02 | 65 | 40 | 0.01 | 8 |
| Progesterone-d9 | pos | 324.2>99.9 | 0.02 | 38 | 25 | 0.01 | 8 |
| Testosterone-d3 | pos | 292.2>109.4 | 0.02 | 35 | 26 | 0.01 | 8 |
| ***Labeled Internal Standard*** | | | | | | | |
| 13C3-Atrazine (ring) | pos | 219.2>177.3 | 0.02 | 30 | 19 | 0.01 |  |
| 13C6-2,4,5-TCPAA (ring) | neg | 258.9>201.2 | 0.02 | 20 | 16 | 0.01 |  |

Analysis of bat tissue extracts from all 4 fractions generated chromatographic raw data that was exceptionally complex due to the presence of a large number of potential interferences. Interferences that mimic the mass spectral response (precursor – product mass transitions) and retention times of target analytes created a potential for false positive results for some of the targeted analytes, and required significant refinement of the conventional identification criteria used to determine the presence of each analyte. Target analyte identification criteria in EPA Method 1694 specifies that analytes elute within 0.25 minutes of the retention time in an associated calibration standard. In this study, a retention window of +/- 0.25 minutes was determined to be too broad to eliminate false positives due to interferences. For the refined identification criteria, we recalculated the expected retention time for each analyte in each sample based on the retention times of the standards, the spikes, and the isotopically-labeled standards spiked into every extract. A retention window of 0.02 minutes relative to the recalculated expected retention time was then applied to the identification of target analytes. Using this more stringent procedure, all of our matrix spike samples and laboratory control samples correctly identified the presence of the analyte of interest. The refined identification process also eliminated substantially more peaks likely to be interferences from the reported sample results than the conventional identification procedures.

Several quality control checks were included as part of the analytical procedure, and these checks are all used to establish the reliability and validity of the results from the bat tissues. The data from these quality control checks are discussed below. Method blanks were included with each set of bat tissue samples in order to determine whether laboratory artifacts could produce false positive results. Analytes that were found in the method blanks required further data interpretation to ascertain whether positive results in the tissue samples were legitimate detections or false positives.

In all three batches one sample was chosen for a matrix spike and matrix spike duplicate analysis. These are actual samples of the bat tissue that are spiked with all of the target analytes. The results from the matrix spike and matrix spike duplicate analyses indicate whether the analytes can be successfully and reproducibly recovered from the bat tissue matrix. Recoveries and method performance were deemed acceptable for a suite of compounds discussed in the Results Section. Data were rejected for compounds for which matrix spike results fell outside of the acceptable recovery range, or for which the relative percent difference (RPD) in matrix spike recoveries violated USEPA Method 1694 QA/QC protocol. If USEPA matrix spike and RPD matrix spike limits were not available, data were rejected if matrix spike recoveries fell outside of a 70 – 150% range or RPD exceeded 45%. Analyte concentrations that did not exceed at least 5 times the concentration detected in the laboratory method blanks were rejected.
