# Supplementary material for: Contaminants of Emerging Concern in Bats from the Northeastern United States
Source: Arch Environ Contam Toxicol. 2015 Aug 6;69(4):411–21. doi: 10.1007/s00244-015-0196-x (PMC4600474; doi:10.1007/s00244-015-0196-x)
Supplement: Supplementary file 2 — Supplementary material 2 (DOCX 27 kb) [file 244_2015_196_MOESM2_ESM.docx]

Table S1. Bat tissue summary of results for contaminants of emerging concern. Analysis by TestAmerica. All results are in ng/g. LOD=limit of detection. USEPA Method 1694 (USEPA 2007). %RPD= relative percent difference between spike recoveries. B=blank flag.

| Analyte | Arithmetic mean conc in 3 method blanks | # Detects | Arithmetic mean conc | Max conc | Min conc | Average LOD | Matrix spike and matrix spike duplicate recovery % | EPA 1694 accepted spike recovery % | RPD % |
| --- | --- | --- | --- | --- | --- | --- | --- | --- | --- |
| 1,7-Dimethylxanthine | ND | 1 | 16 |  |  | 15 | 145 & 141 | 50-138 | 3 |
| 4-Hydroxydiclofenac | 26 | 1 | 169 |  |  | 76 | 63 & 37 |  | 53 |
| Acetaminophen | ND | 0 |  |  |  |  | 37 & 31 | 50-120 | 19 |
| Albuterol | ND | 0 |  |  |  |  | 136 & 127 | 50-133 | 7 |
| Atenolol | ND | 0 |  |  |  |  | 110 & 93 |  | 17 |
| Atorvastatin | 4.6 | 0 |  |  |  |  | 106 & 66 |  | 46 |
| Atrazine | 0.32 | 0 |  |  |  |  | 61 & 82 |  | 30 |
| Azithromycin | ND | 0 |  |  |  |  | 88 & 107 | 33 - 120 | 20 |
| Bisphenol A | ND | 3 | 1294 | 3576 | 76 | 146 | 174&116 |  | 40 |
| Caffeine | ND | 6 | 1509 | 8692 | 6 | 14 | 65&55 | 50-124 | 16 |
| Carbadox | ND | 0 |  |  |  |  | 14 & 15 | 33-144 | 5 |
| Carbamazepine | ND | 0 |  |  |  |  | 111 & 109 | 21-137 | 1 |
| Cimetidine | ND | 0 |  |  |  |  | 84 & 69 | 5-120 | 19 |
| Clarithromycin | 0.14B | 1 | 0.03 |  |  | 0.01 | 43 & 56 | 8-154 | 27 |
| Cloxacillin | ND | 1 | 0.6 |  |  | 0.35 | 53 & 59 | 5-200 | 9 |
| Codeine | ND | 1 | 3.6 |  |  | 5.9 | 0 & 0 | 34-129 |  |
| Cotinine | ND | 5 | 11 | 22 | 3 | 1.7 | 137 & 127 | 50-124 | 7 |
| DEET | 3.8 | 10 | 20 | 63 | 2 | 5.5 | 143 & 142 |  | 1 |
| Diclofenac | ND | 1 | 405 |  |  | 51 | 291 &190 |  | 42 |
| Digoxigenin | ND | 4 | 14 | 37 | 3 | 4.6 | 35 & 32 | 8-183 | 9 |
| Digoxin | ND | 2 | 30 | 45 | 15 | 21 | 188 & 205 | 5-148 | 9 |
| Diltiazem | 0.01 | 4 | 0.07 | 0.11 | 0.02 | 0.02 | 113 & 108 | 11-120 | 4 |
| Diphenhydramine | 0.9 | 14 | 3 | 10 | 1 | 0.36 | 168 & 168 | 48-120 | 0 |
| Equilenin | ND | 1 | 5 |  |  | 4.1 | 97 & 13 |  | 153 |
| Equilin | ND | 3 | 42 | 71 | 10 | 38 | 479 & 284 |  | 51 |
| Erythromycin | 9.2B | 4 | 16 | 22 | 9 | 5.6 | 120 & 192 |  | 46 |
| Estriol | ND | 1 | 2 |  |  | 1.8 | 150 & 103 |  | 37 |
| Estrone | 2.1 | 21 | 44 | 216 | 4 | 18 | 252 & 312 |  | 21 |
| Flumequine | 0.26B | 1 | 0.28 |  |  | 0.13 | 150 & 160 | 36-200 | 6 |
| Fluoxetine | ND | 4 | 19 | 35 | 1 | 3.4 | 179 & 151 | 49-125 | 17 |
| Gemfibrozil | ND | 2 | 1 | 0.7 | 0.6 | 0.03 | 90(87&94) | 50-120 | 8 |
| Hydrocodone | 0.26 | 0 |  |  |  |  | 109 & 114 |  | 4 |
| Ibuprofen | ND | 4 | 4 | 8 | 2 | 1.7 | 102 & 101 | 50-120 | 1 |
| Iopromide | ND | 2 | 79 | 99 | 59 | 14 | 39 & 71 |  | 59 |
| Lincomycin | 0.22 | 1 | 1 |  |  | 0.9 | 101 & 101 | 5-120 | 0 |
| Lorazepam | ND | 1 | 860 |  |  | 480 | 53 & 21 |  | 86 |
| Meprobamate | ND | 1 | 22 |  |  | 12 | 97 & 106 |  | 9 |
| Methadone | ND | 1 | 4 |  |  | 0.6 | 59 & 172 |  | 97 |
| Miconazole | 5.6B | 20 | 6 | 30 | 1 | 2.4 | 7 & 3 | 27 - 120 | 71 |
| Morphine | ND | 0 |  |  |  |  | 91 & 106 |  | 15 |
| Naproxen | ND | 2 | 3 | 4 | 2 | 2.4 | 69 & 52 | 50 - 120 | 28 |
| Nifedipine | ND | 0 |  |  |  |  | 48 & 45 |  | 6 |
| Ormetoprim | ND | 0 |  |  |  |  | 127 & 97 | 50 - 120 | 27 |
| Oxolinic Acid | 2.6 | 7 | 8 | 11 | 4 | 2.1 | 252 & 243 | 42 - 124 | 4 |
| Oxybenzone **^a^** | ND | 5 | 452 | 1226 | 91 | 137 | 62 & 73 |  | 17 |
| Penicillin G | ND | 0 |  |  |  |  | 11 & 34 | 5 - 200 | 103 |
| Penicillin V | ND | 5 | 2 | 3 | 1 | 0.33 | 26 & 36 | 5-200 | 33 |
| Pentoxifylline | ND | 1 | 0.5 |  |  | 0.25 | 106 & 107 |  | 1 |
| Phenytoin | ND | 0 |  |  |  |  | 75 & 58 |  | 26 |
| Primidone | ND | 0 |  |  |  |  | 80 & 45 |  | 56 |
| Progesterone | ND | 0 |  |  |  |  | 0 & 0 |  |  |
| Ranitidine | ND | 3 | 2 | 6 | 0 | 0.6 | 33 & 37 | 24-160 | 11 |
| Roxithromycin | ND | 0 |  |  |  |  | 34 & 24 | 38 - 120 | 34 |
| Salicylic Acid | 6.5 | 23 | 67 | 146 | 18 | 7.5 | 115 & 118 |  | 3 |
| Sildenafil | ND | 1 | 0.3 |  |  | 0.1 | 79 & 76 |  | 3 |
| Sucralose | ND | 0 |  |  |  |  | 0 & 0 |  |  |
| Sulfachloropyridazine | ND | 2 | 4 | 6 | 2 | 4.6 | 91 & 91 | 50-200 | 0 |
| Sulfadiazine | ND | 2 | 14 | 22 | 6 | 7.4 | 490 & 390 | 5-200 | 23 |
| Sulfadimethoxine | ND | 3 | 13 | 25 | 3 | 5.1 | 234 & 188 | 50-120 | 22 |
| Sulfamerazine | ND | 2 | 184 | 363 | 6 | 3.6 | 247 & 238 | 50-148 | 4 |
| Sulfamethazine | ND | 0 |  |  |  |  | 124 & 122 | 50-142 | 2 |
| Sulfamethizole | ND | 1 | 10 |  |  | 8.9 | 97 & 29 | 50-120 | 107 |
| Sulfamethoxazole | ND | 2 | 105 | 208 | 1 | 64 | 166 & 96 | 50-120 | 54 |
| Sulfanilamide | 11.6 | 0 |  |  |  |  | 184 & 172 | 5-189 | 7 |
| Sulfathiazole | 4.1 | 3 | 62 | 102 | 35 | 33 | 103 & 66 | 41-120 | 43 |
| Testosterone | 0.06 | 5 | 6 | 12 | 2 | 2.6 | 87 & 144 |  | 49 |
| Thiabendazole | ND | 13 | 0.5 | 4 | 0 | 0.11 | 111 & 114 | 50-120 | 3 |
| Triclocarban | 0.19 | 19 | 1.3 | 9 | 0 | 0.02 | 100 & 69 | 50-120 | 37 |
| Triclosan | 7.8 | 7 | 41 | 88 | 11 | 3.6 | 63 & 61 | 50-120 | 2 |
| Trimethoprim | ND | 0 |  |  |  |  | 100 & 81 | 50-126 | 21 |
| tris(1,3-dichloro-2-propyl)phosphate (TDCPP) | ND | 0 |  |  |  |  | 101 & 155 |  | 42 |
| tris(1-chloro-2-propyl)phosphate (TCPP) | 6.9 | 20 | 16 | 54 | 4 | 1.1 | 128 & 150 |  | 16 |
| tris(2-chloroethyl)phosphate (TCEP) | 2.7 | 3 | 6 | 7 | 6 | 2.9 | 89 & 108 |  | 20 |
| Tylosin | ND | 1 | 2 |  |  | 0.48 | 94 & 45 | 16-149 | 70 |
| Warfarin | ND | 5 | 86 | 171 | 20 | 7.9 | 66 & 79 | 50-120 | 18 |
| **^a^**  lab determined oxybenzone not detectable with method | | | | | | | | | |
